# Brodie's Abscess: A Diagnostic Conundrum

**DOI:** 10.7759/cureus.16426

**Published:** 2021-07-16

**Authors:** Muhammad Salik, Muhammad Hussain Mir, Deepa Philip, Shobit Verma

**Affiliations:** 1 Trauma and Orthopaedics, Luton and Dunstable University Hospital, Luton, GBR

**Keywords:** brodie's abscess, subacute osteomyelitis, limping child, atraumatic limp, osteo-myelitis, pyogenic osteomylitis

## Abstract

Atraumatic limb pain in a child raises concerns in a medical setting. That is how a typical case of Brodie's abscess presents, having pain without any other symptoms of systemic illness. Assessment and investigations might also not reveal anything significant unless adequate imaging is done. Although Brodie's abscess has a very low rate of complications and morbidity/disability, it is important that such a presentation is assessed clinically with a diagnosis of Brodie's abscess in mind to ensure an uneventful and good outcome. We illustrate in this case report a similar presentation of an 11-year-old girl who had multiple visits to primary care. She was then assessed through radiological imaging. By the time of her diagnosis, her abscess had protruded through the skin. Thankfully management was done swiftly after identification and the final outcome was good with complete recovery.

## Introduction

Brodie’s abscess is defined as a subacute or acute chronic osteomyelitis of the bone. It is an uncommon condition, usually mistaken for being a bone tumor [1], and has been frequently observed to involve the metaphysis of bones (especially tibia) [2]. Making an accurate and timely diagnosis is usually a challenge as pain or swelling are generally the most stereotypical and vague complaints at presentation [3]. Patients usually remain afebrile often with unremarkable inflammatory markers and rarely exhibiting any other signs of systemic illness [3]. Therefore, in the absence of any physiological or hematological signs of illness other than pain, many of these cases can end up being symptomatically treated until definitive testing and management take place [4,5]. Here, we present a similar case with all the hallmark features of Brodie’s abscess. What makes this case especially interesting is that the patient developed an expanding extruding swelling at the site of the original lesion, growing from the source of the infection. The abscess was identified and treated successfully with surgical curettage and drilling, followed by a course of long-term antibiotics. *Staphylococcus aureus* was grown as the causative organism in our case and has been found to be the most prevalent identified pathogen in cases of Brodie’s abscess [6,7].

## Case presentation

**Figure 1:**
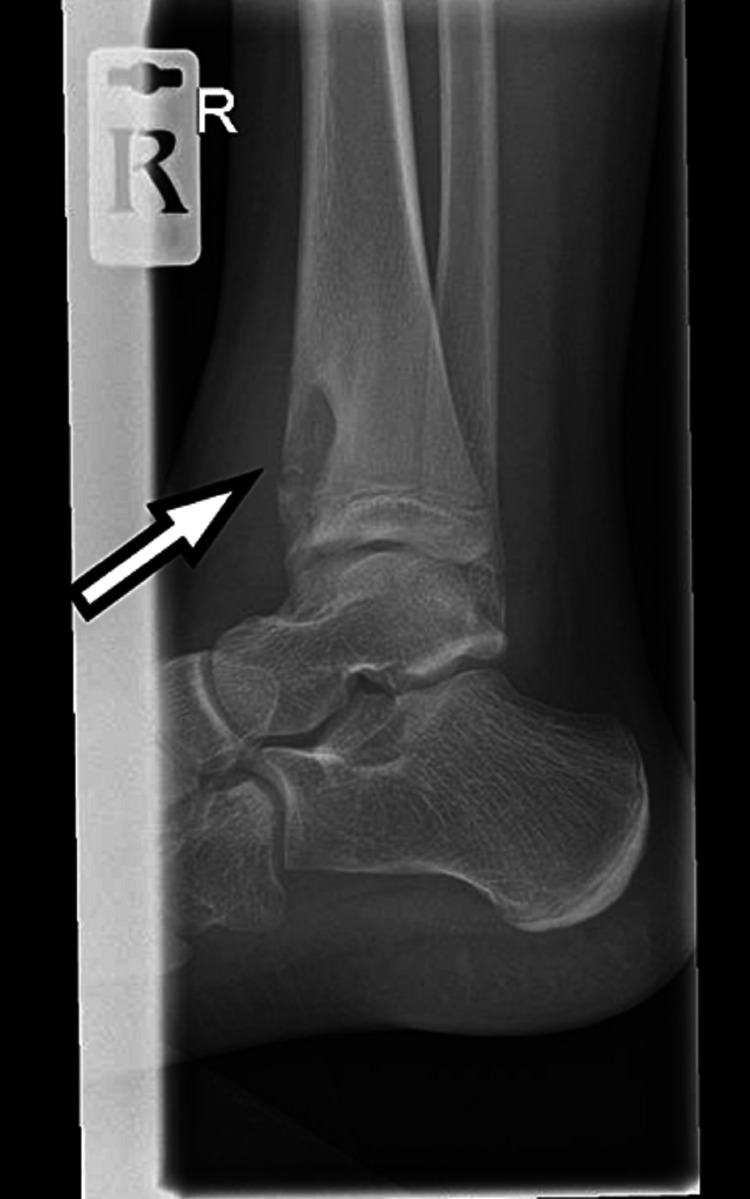
Lateral radiograph showing the lesion in the distal tibia involving the epiphysis, physis, and metaphysis

An 11-year-old girl was referred to the pediatric assessment unit due to a report of a lesion on her distal tibia. The X-ray was performed a few days prior to presentation and was reported and flagged up by the radiologist, which subsequently led to calling the girl in for a review on the weekend. The X-ray showed a lesion extending into the distal tibia (Figure 1).

This X-ray triggered an urgent assessment of the patient by the general practitioner (GP) and an urgent MRI request was made to identify any bone tumors. Following is the chronological history of the case.

**Figure 2:**
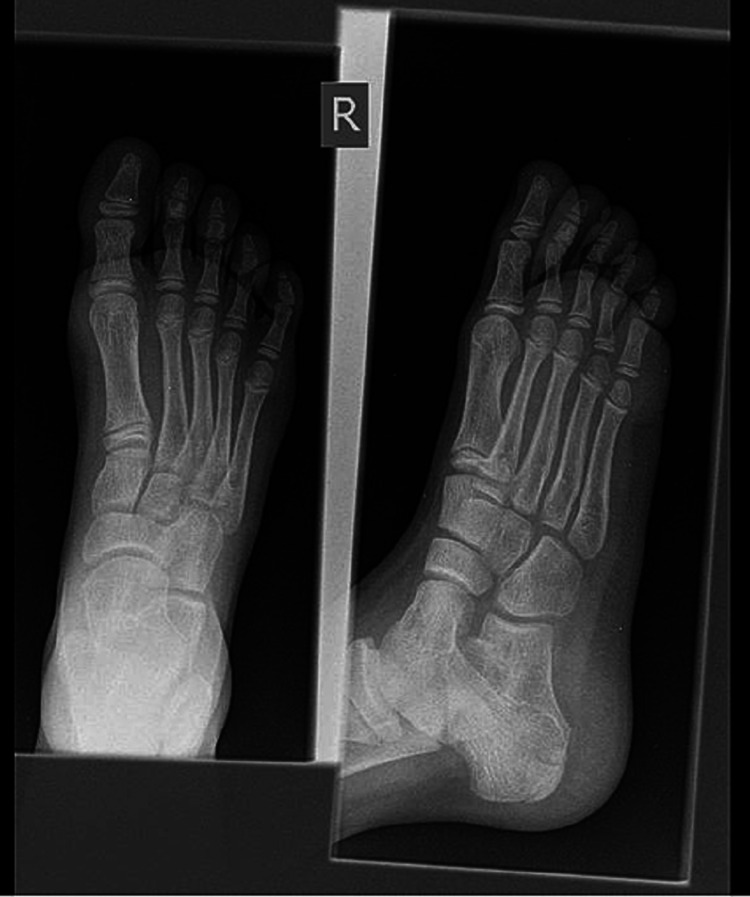
X-ray right foot AP and lateral view done at the time of original presentation having an inadequate view for proper diagnosis as the distal tibia is not visible

The patient originally presented in the accidents and emergency department (A&E) three years ago with the complaint of ankle pain. An X-ray was done at the time of original presentation with only the ankle and the foot visible (Figure 2). No abnormality was detected and the patient was sent home on analgesia.

As seen in the original X-ray above, a complete assessment of the limb is not possible from this image as it does not contain a full view of the affected extremity.

The patient kept on having intermittent complaints of right lower leg pain over the course of two to three years until it exacerbated considerably in the last year leading to multiple visits to the GP. In the absence of any other alarming feature and any preceding or current history of fever, infection, trauma, or superficial skin changes, the pain was managed symptomatically.

**Figure 3:**
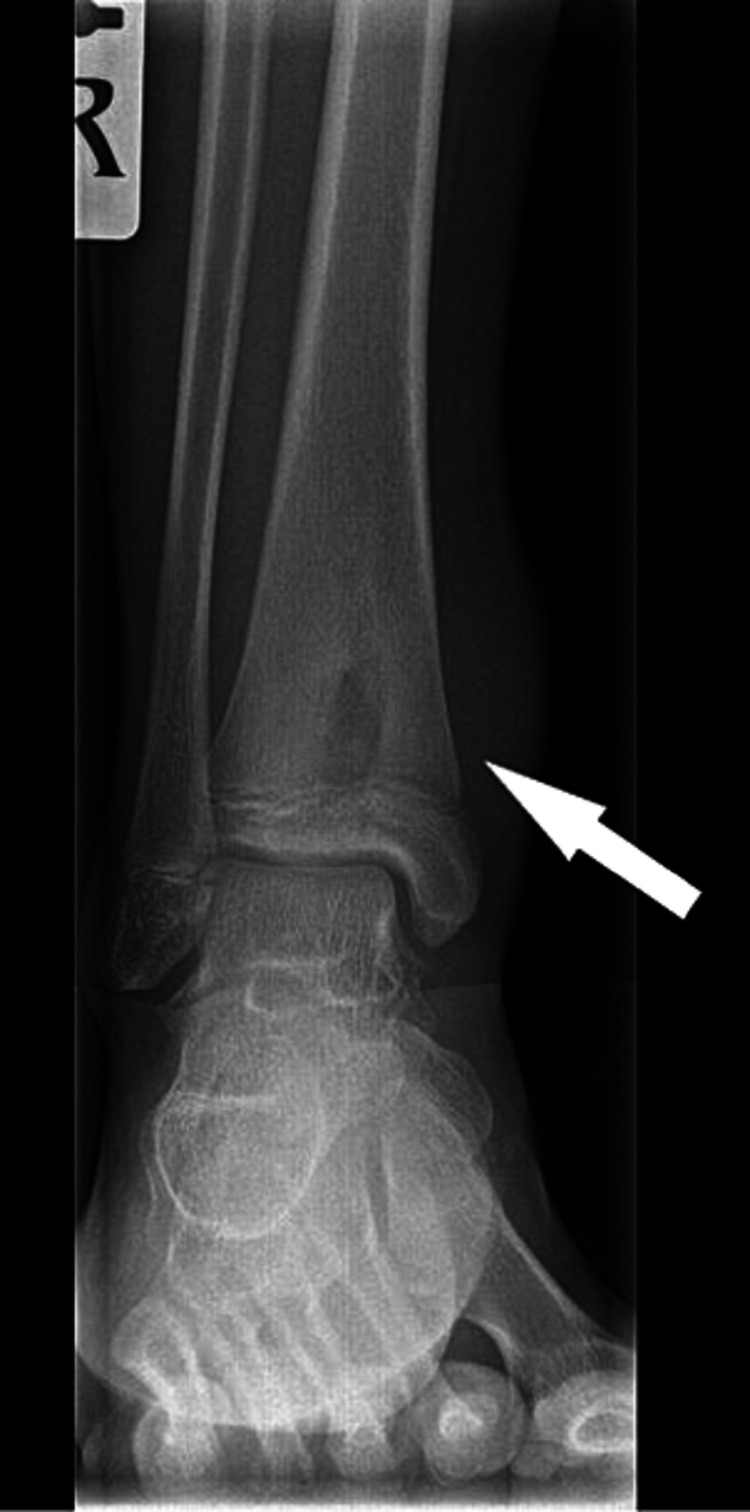
X-ray right distal tibia and fibula AP radiograph showing lesion in the distal tibia involving the physis and metaphysis

The pain continued to worsen despite symptomatic management and a new X-ray was requested. This time, the X-ray included the distal tibia. Following is the radiolucent lesion visible very clearly on the new X-ray (Figure 3).

**Figure 4:**
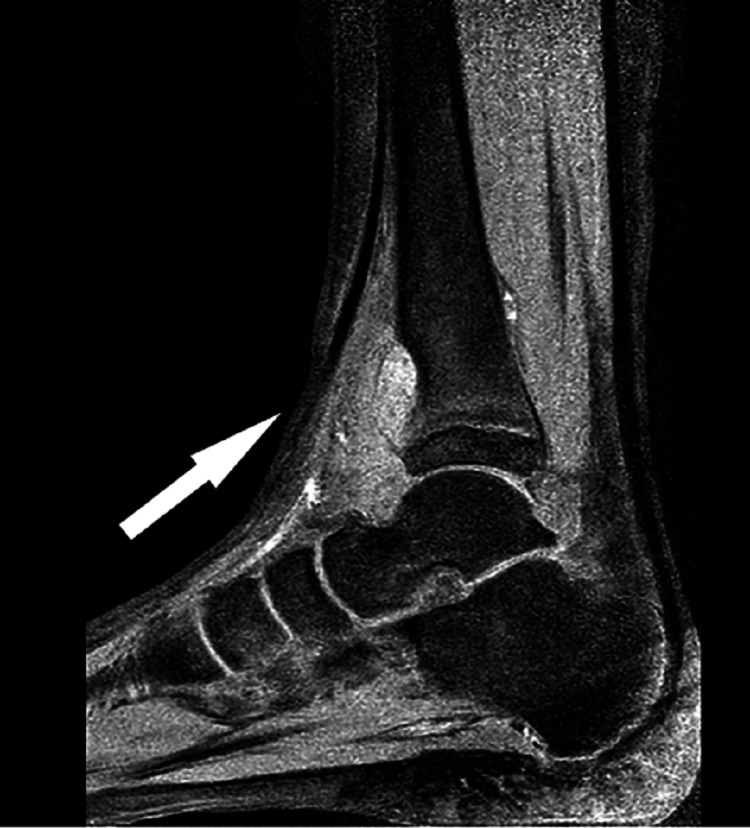
Sagittal view of right lower limb MRI demonstrating an abscess in the distal tibia breaching the cortex and extending into the surrounding soft tissue

**Figure 5:**
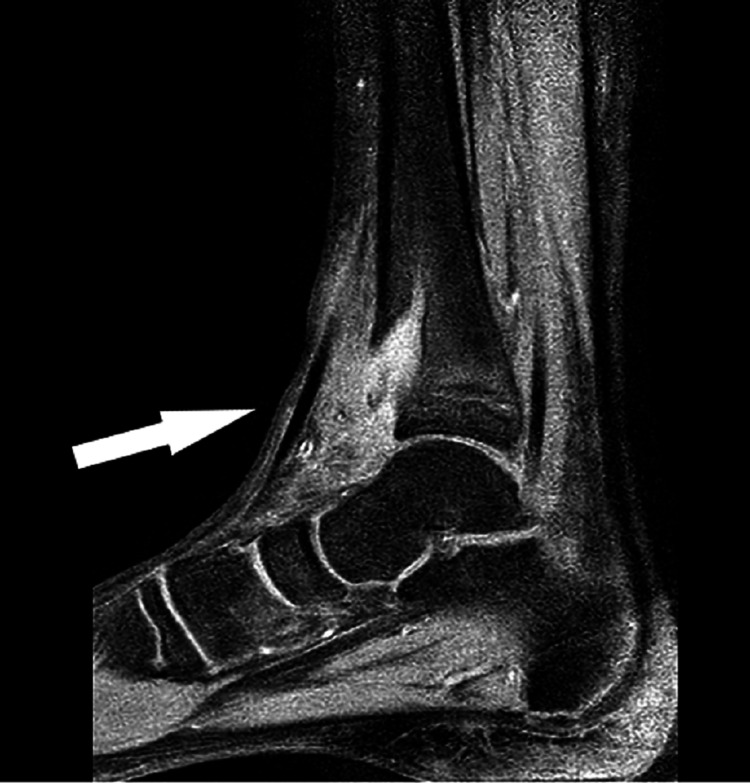
Another MRI image of the same abscess showing involvement of physis and extension into the metaphysis

This alarming image prompted the need for an MRI scan of the lower limb and her case was escalated to the paediatric orthopaedic consultant. Following are the images from the MRI (Figures 4, 5).

The radiologist after reviewing the above images raised concerns of a suspected bone tumor on his report. In light of the report and concerns of the radiologist, the patient was asked to present in the paediatric assessment where she was reassessed. Worsening of the intermittent pain in the lower limb along with mild swelling was noted around the ankle in the history and examination. As mentioned earlier, there was no complaint of fever, and the patient was well otherwise. Blood tests were reviewed which were also normal. However, the new point of concern in her reassessment was that her symptoms had worsened to a point where she had started experiencing difficulty in weight-bearing. Therefore, an urgent referral was made to the regional centre for expert advice regarding diagnosis and further management.

**Figure 6:**
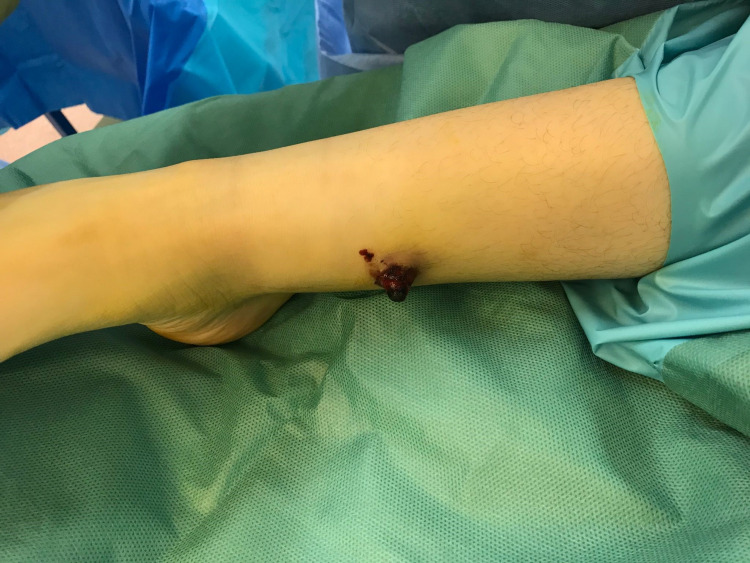
Pre-operative image showing extravasation of the abscess from the skin

**Figure 7:**
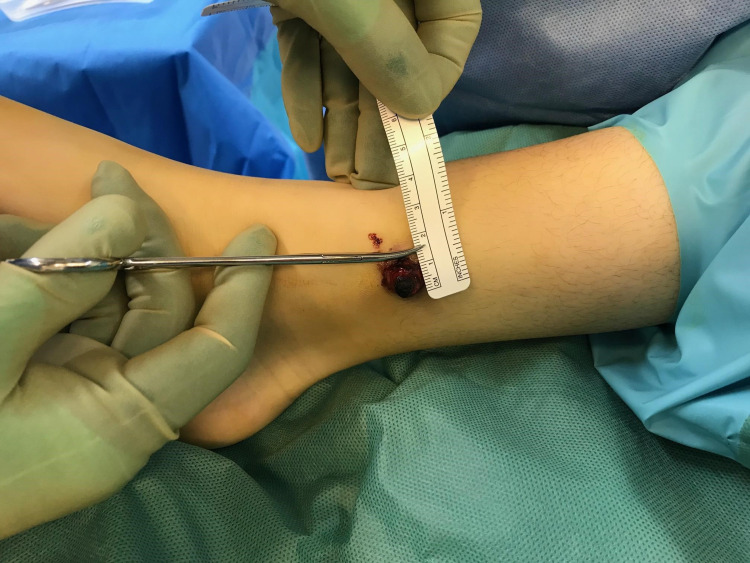
Extravasated Brodie's abscess measuring 1.5cm in diameter

A diagnosis of Brodie's abscess was made and the patient was booked for a semi-elective surgery to treat the abscess. When the patient presented for the surgery, the abscess had extravasated from the skin and was protruding outwards (Figures 6, 7).

**Figure 8:**
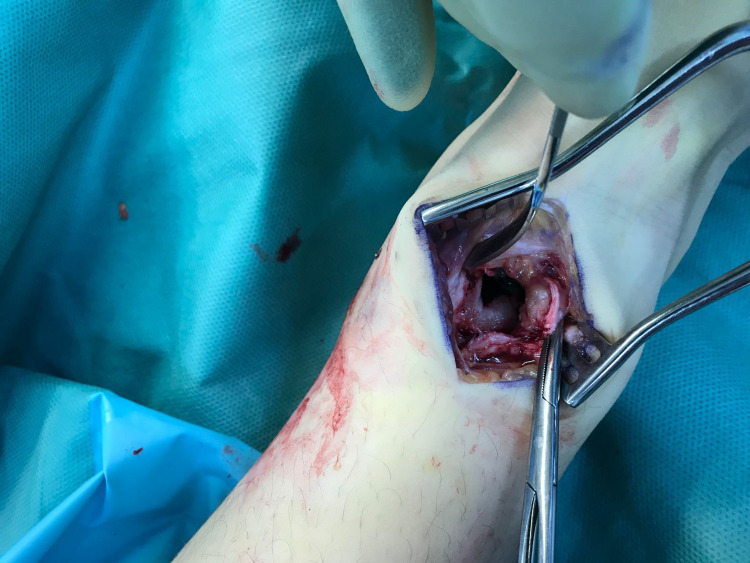
Intra-operative image pre-curettage of Brodie's abscess

The abscess was incised and drained. Intra-operative images are attached below showing the extent of invasion of the abscess into the growing tibia of the 11-year-old girl (Figure 8).

**Figure 9:**
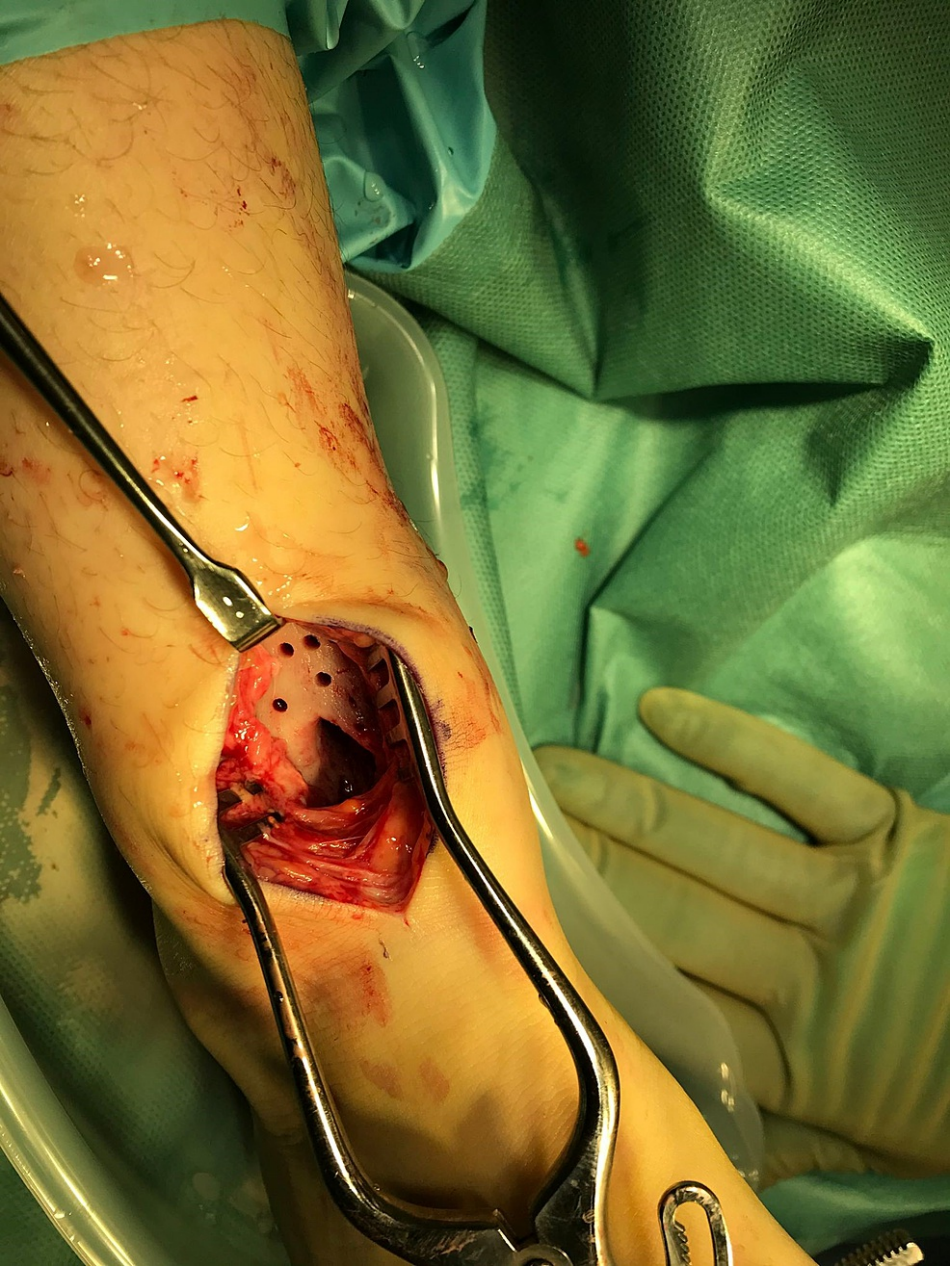
Intra-operative image post-curettage. Holes were drilled to encourage blood supply and antibiotics were sprinkled in the operative site

Holes were drilled in the bone to encourage blood supply. Curettage was done and Vancomycin 1g was instilled in the cavity. The wound was subsequently approximated and closed (Figure 9).

Post-operation recovery remained uneventful and the pain was managed with simple analgesia. She was also started on long-term antibiotics therapy (two weeks of IV followed by two weeks of oral doses) and follow-up was arranged with the paediatric orthopaedic team. Cultures taken intra-operatively grew *Staphylococcus aureus*, which, as mentioned previously, is one of the typical organisms associated with Brodie’s abscess.

**Figure 10:**
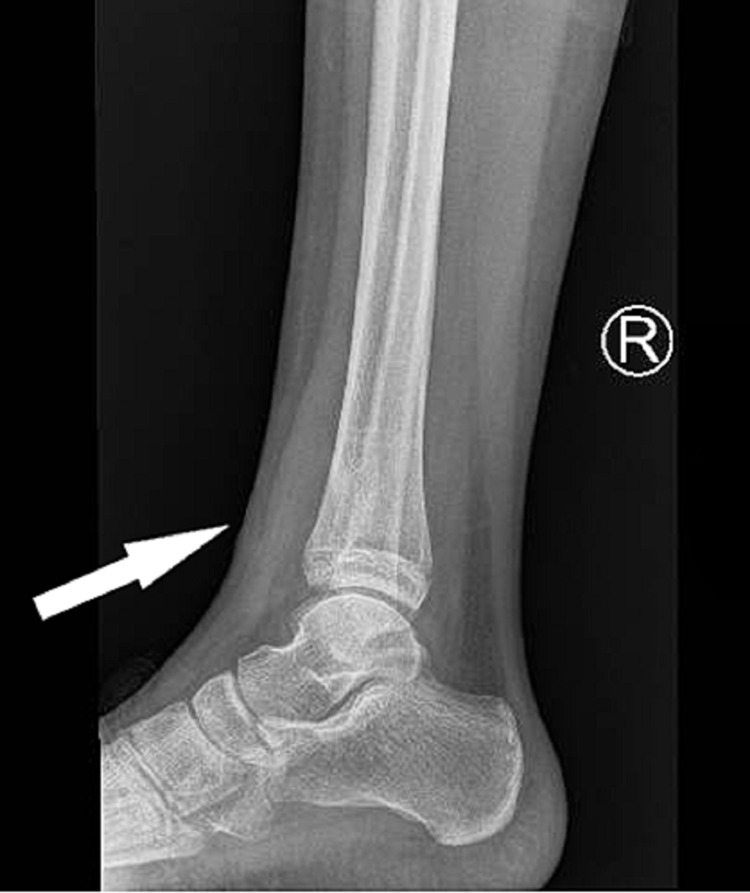
Post-operative X-ray showing good healing at the former site of Brodie's abscess

The patient was seen in the clinic in six weeks where she was assessed to be doing well clinically and also showed evidence of good healing on her new X-rays (Figure 10). 

## Discussion

Brodie’s abscess has an insidious onset and can be difficult to diagnose [5]. X-rays taken at the first presentation should be of good quality and ideally should cover sufficient length of the bone above and below the joint or the area of concern in order to have a proper assessment of the metaphysis, physis, and epiphysis [2]. Although the appearance of the lesion looks alarming, care should be taken in communicating with the patient and family (especially in the case of minors) to not portray this as a definite diagnosis of bone cancer [1]. The treatment options would usually involve surgery followed by long-term antibiotics [3]. Prognosis is generally good with very low chances of recurrence or permanent disability [3].

## Conclusions

It is quite possible for Brodie's abscess to be missed by clinicians due to its infrequent, wide-ranging presentation, and subtle specific or absent systemic symptoms. It is therefore of utmost importance that clinicians evaluate paediatric patients or young adults who present with atraumatic appendage pain carefully to avoid complications and risk of long-term disability and amputation in the aforementioned demographic group.
